# *Rickettsia vini* n. sp. (Rickettsiaceae) infecting the tick *Ixodes arboricola* (Acari: Ixodidae)

**DOI:** 10.1186/s13071-016-1742-8

**Published:** 2016-08-26

**Authors:** Marketa Novakova, Francisco B. Costa, Frantisek Krause, Ivan Literak, Marcelo B. Labruna

**Affiliations:** 1 Department of Biology and Wildlife Diseases, Faculty of Veterinary Hygiene and Ecology, University of Veterinary and Pharmaceutical Sciences Brno, Brno, Czech Republic; 2 CEITEC, University of Veterinary and Pharmaceutical Sciences Brno, Brno, Czech Republic; 3 Department of Preventive Veterinary Medicine and Animal Health, Faculty of Veterinary Medicine, University of São Paulo, São Paulo, Brazil; 4 Breclav, Czech Republic

**Keywords:** Ticks, *Ixodes arboricola*, Tree-hole tick, Ixodidae, Ornitophilic ticks, Nidicolous ticks, *Rickettsia vini*, Rickettsiae, Spotted fever group, Czech Republic

## Abstract

**Background:**

Recently, a new rickettsia named ‘*Candidatus* Rickettsia vini’ belonging to the spotted fever group has been molecularly detected in *Ixodes arboricola* ticks in Spain, the Czech Republic, Slovakia and Turkey, with prevalence reaching up to 100 %. The aim of this study was to isolate this rickettsia in pure culture, and to describe it as a new *Rickettsia* species.

**Methods:**

A total of 148 ornitophilic nidicolous ticks *Ixodes arboricola* were collected in a forest near Breclav (Czech Republic) and examined for rickettsiae. Shell vial technique was applied to isolate rickettsiae in Vero cells. Rickettsial isolation was confirmed by optical microscopy and sequencing of partial sequences of the rickettsial genes *gltA*, *ompA*, *ompB*, and *htrA*. Laboratory guinea pigs and chickens were used for experimental infestations and infections. Animal blood sera were tested by immunofluorescence assay employing crude antigens of various rickettsiae.

**Results:**

*Rickettsia vini* n. sp. was successfully isolated from three males of *I. arboricola*. Phylogenetic analysis of fragments of 1092, 590, 800, and 497 nucleotides of the *gltA*, *ompA*, *ompB*, and *htrA* genes, respectively, showed closest proximity of *R. vini* n. sp. to *Rickettsia japonica* and *Rickettsia heilongjiangensis* belonging to the spotted fever group. Experimental infection of guinea pigs and chickens with *R. vini* led to various levels of cross-reactions of *R. vini*-homologous antibodies with *Rickettsia rickettsii*, *Rickettsia parkeri*, ‘*Candidatus* Rickettsia amblyommii’, *Rickettsia rhipicephali*, *Rickettsia bellii*, and *Rickettsia felis*. Laboratory infestations by *R. vini-*infected *I. arboricola* larvae on chickens led to no seroconversion to *R. vini* n. sp., nor cross-reactions with *R. rickettsii*, *R. parkeri*, ‘*Ca.* R. amblyommii’, *R. rhipicephali*, *R. bellii* or *R. felis*.

**Conclusions:**

Our results suggest that *R. vini* n. sp. is possibly a tick endosymbiont, not pathogenic for guinea pigs and chickens. Regarding specific phenotypic characters and significant differences of DNA sequences in comparison to the most closely related species (*R. japonica* and *R. heilongjiangensis*), we propose to classify the isolate as a new species, *Rickettsia vini*.

**Electronic supplementary material:**

The online version of this article (doi:10.1186/s13071-016-1742-8) contains supplementary material, which is available to authorized users.

## Background

Rickettsiae are Gram-negative coccobacilli belonging to the family Rickettsiaceae, order Rickettsiales in the alpha subdivision of the class *Proteobacteria. Rickettsia* spp. have small genomes (1.1–2.1 Mb) resulted from reductive evolution caused by their obligate endosymbiotic relationship to eukaryotic cells [1]. Their host diversity is remarkably high. Although all valid species are associated with arthropods, novel genotypes have also been identified in annelids, amoebae and plants [2, 3]. A number of *Rickettsia* species can propagate in vertebrates, some of them cause diseases in humans and animals, to which they are transmitted by arthropod vectors such as fleas, lice, mites or ticks. Some species are considered non-pathogenic, and novel *Rickettsia* species reveal to be nearly cosmopolitan [4].

Originally, pathogenic rickettsiae used to be divided into two groups, the typhus group that included *Rickettsia prowazekii* and *Rickettsia typhi*, and the spotted fever group (SFG) composed by about 20 species [5]. The taxonomic position of other rickettsial species has remained unclear because of their genetic anomalies. Due to findings of intriguing variety of rickettsiae in arthropods and molecular analysis of rickettsial plasmids, the genus *Rickettsia* has been reclassified into SFG rickettsiae, typhus group rickettsiae, the transitional group, the *Rickettsia bellii* group, the *Rickettsia canadensis* group, and several basal groups [3, 6]. However, some authors do not support the creation of the transitional group claiming that it is not monophyletic and is unhelpful as it does not take into account epidemiological criteria [1].

Tick-borne rickettsioses are caused by rickettsiae belonging to the SFG [4]. Rapid development of molecular methods brought reversed approach to tick-borne pathogen research, when disease cases are detected years after the tick-borne microorganism was first discovered [7]. There have been species of rickettisae detected in ticks years or decades before they became associated with human illness cases, e.g. *Rickettsia monacensis*, *Rickettsia parkeri*, *Rickettsia massiliae* and *Rickettsia slovaca* [4, 8]. It is not clear if these novel tick-borne diseases were not noticed by physicians or whether they were absent. While it has been suggested that any novel described rickettsia from ticks should be considered a potential pathogen [5], many tick species just do not bite humans under natural conditions, or some rickettsial agents are just tick endosymbionts.

Recently, a novel SFG rickettsia has been found by molecular methods in bird-associated ticks. It was named ‘*Candidatus* Rickettsia vini’ and until now it has been detected in Spain, the Czech Republic, Slovakia and Turkey [9–11]. This bacterium has been molecularly detected mainly in *Ixodes arboricola* ticks, in which the prevalence is high (reaching 90–100 %) [11, 12]. It has rarely been found in immature stages of *Ixodes ricinus* [9]. *I. arboricola* tick is widely distributed in the Palaearctic region. It lives in tree holes and nest boxes where it feeds on hole-breeding birds. Although this tick species does not represent a primary risk for humans, it shares several host species and overlaps in feeding period with *Ixodes ricinus* [13]. Therefore, tick-borne microorganisms, including ‘*Ca.* R. vini’, could be potentially bridged between these two tick species via co-feeding. Phylogenetic analysis based on partial sequences of four rickettsial genes (*gltA*, *ompA*, *ompB*, *sca4*) showed that ‘*Ca.* R. vini’ segregated closest to *Rickettsia heilongjiangensis* and *Rickettsia japonica*, supported by high bootstrap values [14]. The latter two species are causative agents of the Far East spotted fever (*R. heilongjiangensis*) and the Japanese spotted fever (*R. japonica*), and both have been reported in Asia [4].

In order to describe ‘*Ca.* R. vini’ as a new species, we isolated the bacterium in cell culture for the first time, and performed both molecular and phenotypical characterization of the isolates.

## Methods

### Field study in Breclav, Czech Republic

Free-living *I. arboricola* ticks were collected manually from nest boxes during after-breeding season in Breclav, Czech Republic (48°43'N, 16°54'E, 150 m above sea level, an oak-ash flood-plain forest), an area attractive to tourists. Nesting bird species had been previously identified during the breeding season using a bird guide book [15] and confirmed according to characteristic appearance of the nest during tick collecting. Ticks were identified to species according to Nosek & Sixl [16]. Collected ticks were brought alive to the laboratory and incubated at 12 °C (relative humidity of 80 %) for 3 months and then at 24 °C (relative humidity of 80 %) for 7 days before being subjected to the hemolymph test.

### Hemolymph test and isolation of rickettsiae

Selected individuals were tested for the presence of *Rickettsia*-like structures using the hemolymph test [17]. Shortly, the distal part of a tick leg was cut, then a drop of hemolymph was dried on a microscope slide and stained using Gimenez method [18]. The whole-body remnants were immediately stored at -80 °C to preserve living rickettsial organisms.

Isolation of rickettsiae from the tick samples was performed according to previous protocols [19] with some modifications. Briefly, ticks were surface-sterilized by immersion in iodine-alcohol for 10 min, washed in sterile water, macerated, and resuspended in 600 μl of brain heart infusion (BHI). For each tick sample, two shell vials with a confluent monolayer of Vero cells were each inoculated with 300 μl of the BHI suspension and then centrifuged for 1 h at 700× *g* and 22 °C. The monolayers were washed and fed with 1 ml of minimal essential medium supplemented with 5 % of bovine calf serum (Hyclone, Logan, UT, USA) and 1 % of antibiotics and antifungal (penicillin, streptomycin and amphotericin B) and incubated at 28 °C. Every 3 days, the medium was replaced by a new medium (without antibiotics and antifungal additives), and the aspirated medium was checked for the presence of *Rickettsia*-like organisms by Gimenez staining. If the result was positive, the monolayer of the shell vial was harvested and inoculated into a 25 cm^2^ flask containing a monolayer of confluent uninfected Vero cells. Cells in the 25 cm^2^ flask were checked by Gimenez staining until > 90 % of them were infected, when they were harvested and inoculated into 75 cm^2^ flasks of Vero cells. The level of infection of cells was monitored by Gimenez staining of scraped cells from the inoculated monolayer. The rickettsial isolate was considered to be established in the laboratory after at least three passages through 75 cm^2^ Vero cell flasks, each achieving a proportion > 90 % of infected cells [20].

### Experimental infestations and inoculations

Selected larvae obtained from one egg cluster of *I. arboricola* were PCR-tested to confirm the presence of rickettsial DNA. Then, three tick naive chickens (denoted A, B and C) were each infested with 100 *I. arboricola* larvae from this cluster. Blood samples were collected from the chickens at the beginning of the infestation (day 0) and 21 days later. Two chickens (denoted D, E) and two male guinea pigs (denoted A, B), all tick naive, were each inoculated intraperitoneally with a suspension of ≈ 1 × 10^6^ Vero cells infected with an *I. arboricola* rickettsial isolate derived from a fresh culture containing > 90 % infected cells. Blood samples were collected at 0 and 21 days after inoculation. The guinea pigs were examined daily for fever (if the rectal temperature was > 39.5 °C) and scrotal reactions.

### Serological tests

Animal blood sera were individually tested by immunofluorescence assay (IFA) as described [21], employing crude antigens of five SFG rickettsiae (the *I. arboricola* rickettsial isolate Rv-M1A strain Breclav; *Rickettsia rickettsii* strain Taiaçu [22]; *R. parkeri* strain At24 [23]; ‘*Candidatus* Rickettsia amblyommii’ strain Ac37 [19]; and *Rickettsia rhipicephali* strain HJ5 [24]); a basal group rickettsia (*R. bellii* strain Mogi [22]); and one transitional group rickettsia (*Rickettsia felis* strain Pedreira [25]), which were prepared using whole infected Vero or C6/36 cells as previously described [21]. Sera were diluted in 2-fold increments, beginning from a 1:64 dilution, tested with fluorescein isothiocyanate-labeled rabbit anti-guinea pig IgG (Sigma-Aldrich, St. Louis, MO, USA) or anti-bird IgG-FITC conjugate (Alpha Diagnostic Intl Inc., San Antonio, TX, USA). Endpoint titers for both homologous and heterologous reactions were determined.

### Molecular characterization

All whole-body remnants of the ticks used to inoculate shell vials and infected Vero cell 1st–4th passages were subjected to DNA extraction using the guanidine isothiocyanate technique, as described elsewhere [26], and DNA extracts were stored at -20 °C until they were used as templates for polymerase chain reaction (PCR). DNA samples were tested by a battery of PCR protocols targeting fragments of four rickettsial genes: citrate synthase gene (*gltA*), the 190-kDa outer membrane protein gene (*ompA*), the 120-kDa outer membrane protein gene (*ompB*), and the 17-kDa protein gene (*htrA*) (Table 1). Each PCR run included a negative control (2.5 μl of water) and a positive control (2.5 μl of DNA of *Rickettsia parkeri* strain NOD) samples. PCR products were purified by ExoSAP-IT® (USB), DNA-sequenced by Sanger dideoxy sequencing, and analyzed using BLAST [27] to determine similarities to other *Rickettsia* spp. available in GenBank, National Center for Biotechnology Information (NCBI) [28]. The DNA sequences obtained from the 4th passage-infected cells were submitted to the GenBank database (see below). Phylogenetic analyses were performed using the program MEGA version 6.06 in November 2015 [29]. The newly-generated partial DNA sequences (*gltA*, *ompA*, *ompB*, and *htrA* genes) were analyzed separately, and also concatenated. In both cases, nucleotides were aligned with the corresponding sequences of other *Rickettsia* species available in the GenBank database using MUSCLE algorithm as implemented in MEGA. The resulted alignment was checked and manually corrected. The evolutionary history was inferred using the Maximum Likelihood method based on the Tamura 3-parameter (I + G) model with 1000 replicates of random-addition taxa and tree bisection and reconnection branch swapping. All positions were weighted equally.

**Table 1:** Primer pairs used for amplification of rickettsial genes

Target gene, primer pair no., primer name		Specifity	Sequence	Amplified fragment (nt)	Reference
*gltA*		Genus *Rickettsia*			
1	CS-78		5'-GCAAGTATCGGTGAGGATGTAAT-3'	401	[19]
	CS-323		5'-GCTTCCTTAAAATTCAATAAATCAGGAT-3'		[19]
2	CS-239		5'-GCTCTTCTCATCCTATGGCTATTAT-3'	834	[38]
	CS-1069		5'-CAGGGTCTTCGTGCATTTCTT-3'		[38]
*ompA*		Spotted fever group (SFG)			
3	Rr190.70p		5'-ATGGCGAATATTTCTCCAAAA-3'	632	[39]
	190-701		5'-GTTCCGTTAATGGCAGCATCT-3'		[40]
*ompB*		Genus *Rickettsia* ^a^			
4	120-M59		5'-CCGCAGGGTTGGTAACTGC-3'	820	[41]
	120-807		5'-CCTTTTAGATTACCGCCTAA-3'		[41]
*htrA*		Genus *Rickettsia*			
5	17k-5		5'-GCTTTACAAAATTCTAAAAACCATATA-3'	549	[38]
	17k-3		5'-TGTCTATCAATTCACAACTTGCC-3'		[38]

^a^Except for some species of basal groups (e.g. *Rickettsia bellii*)

### Morphology by light microscopy

Gimenez stained hemolymph smears were examined under oil immersion (at magnification of × 1000; 10× ocular and a 100× objective). Images of *Rickettsia*-like structures and adjacent Vero cells were captured using Leica Microscope DM4000-B.

## Results

### Family Rickettsiaceae Pinkerton 1936

### Genus *Rickettsia* da Rocha-Lima 1916

***Rickettsia vini*n. sp.**

***Type-host***: Tree-hole tick, *Ixodes arboricola* Schultze & Schlottke, 1930 (Acari: Ixodida: Ixodidae).

***Type-locality***: Breclav, Czech Republic.

***Other localities***: La Rioja, Spain; Kızılırmak Delta, Samsun, Turkey; Velky Kosir, Czech Republic; Ziar nad Hronom, Slovakia.

***Type-strain***: The type-strain Breclav^T^ from three *I. arboricola* male ticks, sampled in Breclav, Czech Republic (48°43'N, 16°54'E) in a nest box of *Ficedula albicollis*, is deposited at the Rickettsial Collection of the Laboratory of Parasitic Diseases of the Faculty of Veterinary Medicine, University of São Paulo, São Paulo, Brazil (culture collection codes: Rv-M1A-3P; Rv-M2B-3P; Rv-M3B-3P), and the Rickettsial Collection of the Rickettsial Zoonoses Branch of the Centers for Disease Control and Prevention (CDC), Atlanta, GA, USA (culture collection codes: Rv-M1A-2P; Rv-M2B-2P; Rv-M3B-2P).

***Vector***: Unknown.

***Representative DNA sequences***: GenBank: partial sequences KT187394 (*gltA* gene); KT326194 (*ompA* gene); KT187395 (*ompB* gene); KT187396 (*htrA* gene).

***Etymology***: The name *vini* has been proposed by Palomar et al. [9] who first detected molecularly this bacterium at La Rioja, a vineyard region in Spain. District of Breclav, the type-locality, is also an important area of vine production in the Czech Republic.

### Description

**Fig. 1:**
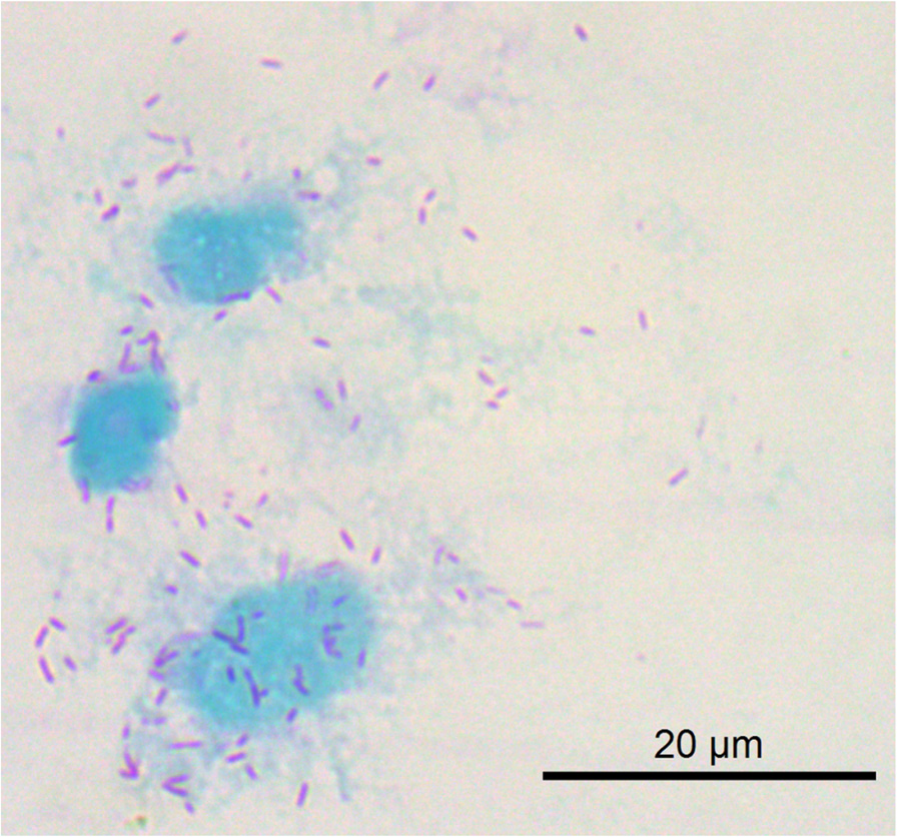
Vero cells infected by *Rickettsia vini* n. sp. strain Breclav visualized by Gimenez staining

*Rickettsia vini* n. sp. is a Gram-negative, nonmotile, obligately intracellular bacterium. The organism has a typical bacillary morphology with binary fission. It grows at 28 °C on Vero cells in minimal essential medium with 5 % bovine calf serum supplement (Fig. 1). Sequencing of *gltA*, *ompA*, *ompB*, and *htrA* genes implies that this bacterium is significantly different from all recognized rickettsial species. It belongs to the SFG and is most closely related to *R. japonica* and *R. heilongjiangensis. Rickettsia vini* n. sp. is not pathogenic for chickens and guinea pigs through intraperitoneal inoculation, although it induces seroconversion in these animals (see below). The pathogenicity of this bacterium for vertebrate hosts, including humans, remains to be elucidated.

### Field study

A total of 35 nest boxes (32 of *Ficedula albicollis*, 3 of species of Paridae) were checked for the presence of ticks. One hundred forty-eight *I. arboricola* ticks (2 engorged nymphs, 18 males, 128 engorged females) were found in 9 (25.7 %) out of 35 nest boxes; all 9 boxes belonged to *F. albicollis* (arithmetic mean ± standard error: 16.4 ± 41.2 ticks per infested nest box). Although one *F. albicollis* nest box contained 18 male and 115 engorged female ticks, all 6 nestlings successfully fledged, which was documented during breeding season. *Ixodes arboricola* females were each stored in a separate tube and egg clusters were laid by 24 of them. Hundreds of larvae hatched after one month.

### Isolation of *Rickettsia vini* n. sp.

All 18 male ticks were subjected to the hemolymph test; of these three were found positive for *Rickettsia*-like organisms within their hemocytes, and subsequently subjected to the isolation of rickettsiae by the shell vial technique. Rickettsiae were successfully isolated from all three ticks and established in Vero cell culture (Fig. 1). The three isolates were designated as Rv-M1A, Rv-M2B, and Rv-M3B.

### Experimental infestations, inoculations and serological tests

The chickens A, B and C that were infested with *R. vini-*infected *I. arboricola* larvae remained seronegative to all seven *Rickettsia* species, including *R. vini* antigens (Table 2). A total of 15 to 17 engorged larvae were recovered from each chicken. Conversely, the chickens D, E that were inoculated with *R. vini-*infected Vero cells showed seroconversion for *R. vini* n. sp., *R. rickettsii*, and ‘*Ca.* R. amblyommii’ with titers ranging from 64 to 512 at 21 days after inoculation. Chicken E also seroconverted to *R. bellii* (Table 2). None of the above five chickens showed apparent signs of disease during the present study.

**Table 2:** Homologous and heterologous endpoint titers of IgG to seven *Rickettsia* species in animal sera

Species	Chickens infested with *R. vini-*infected *Ixodes arboricola*			Animals inoculated with *R. vini* culture			
	Chicken A	Chicken B	Chicken C	Chicken D	Chicken E	Guinea pig A	Guinea pig B
*Rickettsia vini* n. sp.	–^a^	–	–	128	512	512	128
*Rickettsia rickettsii*	–	–	–	64	64	512	–
*Rickettsia parkeri*	–	–	–	–	–	–	–
‘*Ca.* Rickettsia amblyommii’	–	–	–	128	64	256	–
*Rickettsia rhipicephali*	–	–	–	–	–	–	NT
*Rickettsia bellii*	–	–	–	–	256	–	–
*Rickettsia felis*	–	–	–	–	–	–	–

^a^–, negative at the 1:64 serum dilution

*Abbreviation*: *NT* not tested

The guinea pig A showed seroconversion to *R. vini*, *R. rickettsii* and ‘*Ca.* R. amblyommii’ with 512, 512 and 256 endpoint titers, respectively, 21 days after intraperitoneal inoculation of *R. vini-*infected Vero cells. The guinea pig B seroconverted only to *R. vini* n. sp., with a 128 endpoint titer (Table 2). None of these guinea pigs developed fever, scrotal reactions or any other clinical alteration.

### Genotyping and phylogenetic analysis

DNA of infected cells from the 1st to 4th rickettsial passages of all three isolates were tested by PCRs targeting the *gltA*, *ompA*, *ompB*, and *htrA* genes, and 1092, 590, 800, and 497 nucleotides (nt), respectively, of the PCR products were sequenced from each isolate. Sequences obtained from different passages of all three isolates were 100 % identical. BLAST analysis of the *gltA* partial sequence showed 100 % (1092/1092 nt) similarity to the corresponding sequence of two strains of *R. vini* from the Czech Republic and Spain (KJ626330, JF803266) [11, 14]. The *ompA* partial sequence revealed 100 % (590/590 nt) similarity to the corresponding sequence of *R. vini* from Spain (JF758828) [14]. The *ompB* partial sequence showed 99.3 % (764/769 nt) similarity to the corresponding sequence of *Rickettsia* sp. HIR/D91 (KC888953). The *htrA* partial sequence was 99.2 % (493/497 nt) similar to the corresponding sequence of various strains of *R. rickettsii* (AY281069, CP000766, CP000848, CP003305, CP003306, CP003307, CP003309, CP003311, CP003318, CP006009, CP006010, M28479) and *Rickettsia philipii* (CP003308). Before this study, there were no corresponding sequences of the *ompB* and the *htrA* gene fragments of *R. vini* in GenBank.

**Fig. 2:**
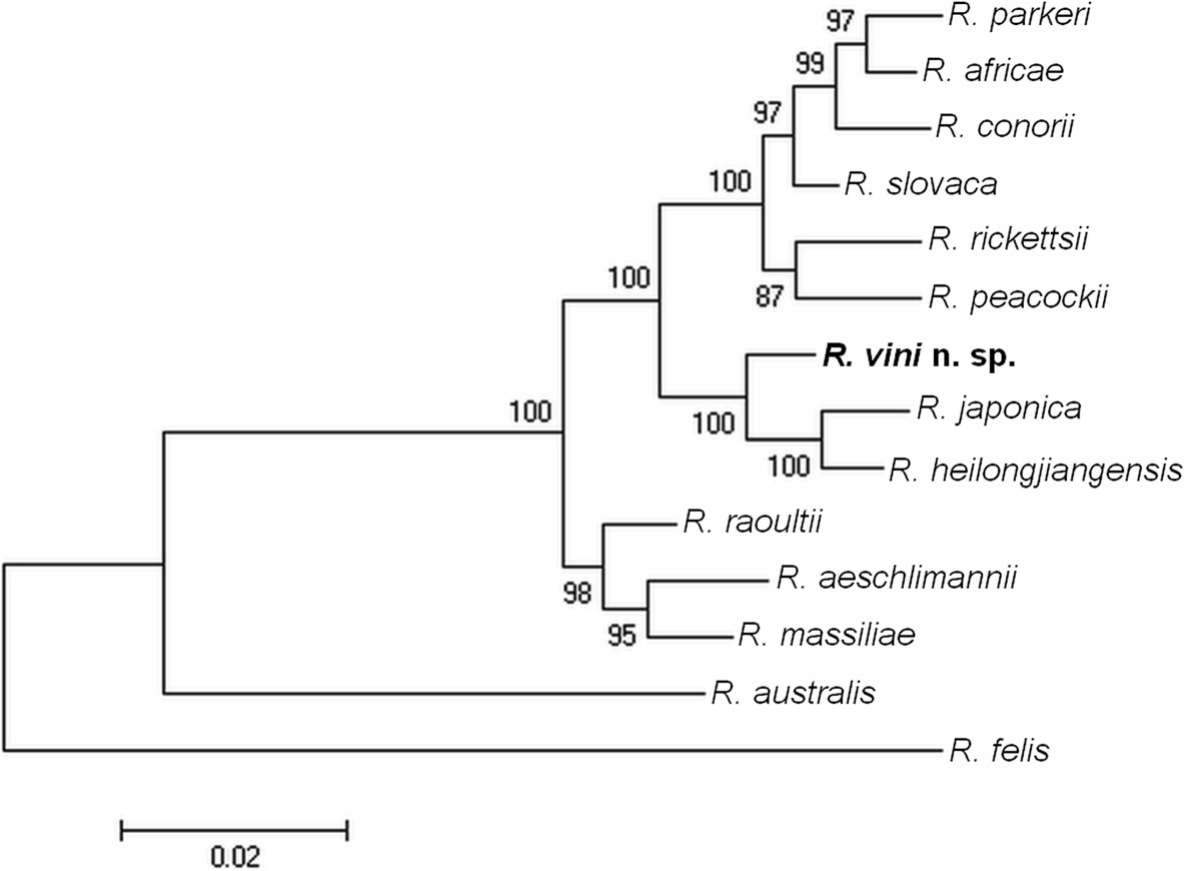
Molecular phylogenetic analysis of *Rickettsia vini* n. sp. isolated from the tick *Ixodes arboricola* (Czech Republic). A total of 2979 unambiguously aligned nucleotide sites of the rickettsial genes *gltA*, *ompA*, *ompB*, and *htrA* were concatenated and subjected to analysis by the Maximum Likelihood method. The bootstrap values obtained by 1000 replicates are shown at the nodes. The tree is drawn to scale; scale-bar indicates nucleotide substitutions (%) per site. The GenBank accession numbers of the sequences included in this analysis are shown in Additional files 1, 2, 3 and 4

Phylogenetic analyses were inferred from the *gltA*, *ompA*, *ompB*, and *htrA* partial sequences, with each gene analyzed separately (Additional files 1, 2, 3 and 4). Then an analysis of a concatenated dataset was carried out on an alignment that included 2979 nt (1092, 590, 800, 497 nt for the *gltA*, *ompA*, *ompB*, and *htrA* genes, respectively). In all analyses, *R. vini* n. sp. segregated closest to *R. japonica* and *R. heilongjiangensis* cluster, which was supported by high bootstrap values (Fig. 2).

## Discussion

This study described and characterized a new species of *Rickettsia*, *R. vini* n. sp., isolated from *I. arboricola* ticks collected in nest-boxes in the Czech Republic. This bacterium was first detected by PCR in *I. arboricola* and *I. ricinus* immature ticks collected from birds in La Rioja, a vineyard region in Spain [9] and named ‘*Ca.* R. vini’ [14]. To date, this bacterium has been detected molecularly in ticks in Europe and Turkey. The Palaearctic distribution of the type-species, the tick *I. arboricola* predicates possibly a similar wide occurrence of *R. vini* n. sp.

Through molecular analyses (PCR detection), infection rates of *R. vini* in *I. arboricola* ticks up to 100 % have been reported [10, 11, 14]; however, we found only three out of 18 males positive for *Rickettsia*-like organisms using the hemolymph test. This may be caused by higher sensitivity of PCR detection, when compared to the hemolymph test, or/and because not all *R. vini-*infected ticks contain rickettsiae in their hemolymph. All PCR-tested unfed larvae of *I. arboricola* from this study contained rickettsial DNA, indicating transovarial transmission of the rickettsial agent.

All animals inoculated intraperitoneally seroconverted after 21 days, sometimes with high homologous antibody titers to *R. vini* n. sp. (Table 2). The guinea pig A showed cross-reactivity for *R. rickettsii* and ‘*Ca.* R. amblyommii’ antigens, while the guinea pig B reacted only to the homologous antigens. Both chickens D and E inoculated with *R. vini-*infected Vero cells showed homologous titers always equal or higher than heterologous titers. Cross-reactivities were observed with closely related species belonging to the SFG such as *R. rickettsii* and ‘*Ca.* R. amblyommii’, although chicken E also reacted to *R. bellii,* a non-SFG agent. Cross-reactivity with lower titres for heterologous antigens has also been observed in experimental studies with guinea pigs intraperitoneally inoculated by Vero cells infected with *R. monteiroi*, *R. bellii*, *R. rickettsii* or *R. canandensis* [30]. Although *R. felis* is phylogenetically closer than *R. bellii* to *R. vini*, no cross-reactivity with *R. felis* was observed*.* These results indicate that *R. vini* n. sp. possibly shares numerous antigenic constituents with other *Rickettsia* species, especially SFG members (Fig. 2). These findings are consistent with previous studies with mice, guinea pigs, dogs and opossums that were inoculated with different *Rickettsia* species [31–34].

Absence of clinical signs in *R. vini-*inoculated chickens D, E and guinea pigs A, B suggests that *R. vini* n. sp. is not pathogenic for these animals. None of the three chickens A, B, C infested by *R. vini-*infected larvae seroconverted, in contrary to chickens D, E that were inoculated with *R. vini* culture. While these results suggest a tick-symbiotical nature of *R. vini*, it is also possible that chickens (and guinea pigs) are just not susceptible to *R. vini.* If this is the case, the seroconversion of inoculated animals in the present study could be just a result of direct contact with bacterial antigens, rather than active infection. Such assumptions need to be confirmed in further studies. Finally, the non-susceptibility of chickens in the present study could be linked to the inoculation route (intraperitoneal inoculation), since other rickettsial agents were shown to cause skin lesions through intradermal inoculations of experimental animals, in contrast to no clinical alterations when the same agents were intraperitoneally inoculated [35, 36]. Moreover, our results of animal inoculations do not exclude a possible susceptibility of the bird hosts of *I. arboricola* to *R. vini* under natural conditions.

The phylogenetic analyses of four rickettsial genes showed that *R. vini* n. sp. belongs to the SFG and is most closely related to *R. japonica* and *R. heilongjiangensis*, which is in compliance with previous studies [11, 14]. In this study, we amplified different and longer fragments of the *ompB* and *htrA* genes of *R. vini* that have not been previously published. *R. japonica* and *R. heilongjiangensis* are associated with various tick vectors and mammal reservoirs [4]. It has been proposed that a new *Rickettsia* species should not show > 99.9 %, > 99.2 %, and > 98.8 % similarity for the *gltA*, *ompB*, and *ompA* genes, respectively, with the most homologous validated species [37]. Similiarity values of *R. vini* DNA sequences with the most closely related validated species are 99.7 % for the *gltA* gene of *R. heilongjiangensis* (CP002912), 96.8 % for the *ompB* gene of *R. japonica* (AP011533), and 97.1 % for the *ompA* gene of *R. heilongjiangensis* (CP002912). These comparison values also support the recognition of *R. vini* as a new species.

## Conclusions

Here we report the first isolation of ‘*Ca.* R. vini’ in cell culture, both molecular and morphological characterization of the isolates, experimental inoculation of laboratory guinea pigs and chickens, and experimental infestation of chickens with *R. vini-*infected ticks. We conclude that *R. vini* n. sp. is not pathogenic for chickens and guinea pigs, although direct inoculation of these animals with *R. vini* resulted in seroconversion. The new species described here is ecologically, geographically and molecularly distinct from any closely related validated species.

## Additional files

Additional file 1: Maximum Likelihood phylogenetic tree based on the partial *gltA* gene including a sequence for *Rickettsia vini* n. sp. (DOCX 637 kb) Additional file 2: Maximum Likelihood phylogenetic tree based on the partial *ompA* gene including a sequence for *Rickettsia vini* n. sp. (DOCX 698 kb) Additional file 3: Maximum Likelihood phylogenetic tree based on the partial *ompB* gene including a sequence for *Rickettsia vini* n. sp. (DOCX 639 kb) Additional file 4: Maximum Likelihood phylogenetic tree based on the partial *htrA* gene including a sequence for *Rickettsia vini* n. sp. (DOCX 522 kb)
