# Supplementary material for: Rickettsia vini n. sp. (Rickettsiaceae) infecting the tick Ixodes arboricola (Acari: Ixodidae)
Source: Parasit Vectors. 2016 Aug 26;9(1):469. doi: 10.1186/s13071-016-1742-8 (PMC5000463; doi:10.1186/s13071-016-1742-8)

**Additional file 3:** Maximum Likelihood phylogenetic tree based on the partial *ompB* gene including a sequence for *Rickettsia vini* n. sp.

**Introduction to Additional file 3:** A total of 800 unambiguously aligned nucleotide sites of the rickettsial gene *ompB* were subjected to a Maximum Likelihood analysis. The evolutionary history was inferred based on the Tamura 3-parameter (I + G) model. The bootstrap values obtained by 1,000 replicates are shown at the nodes; only bootstrap values ≥ 50% are shown. All positions were weighted equally. The tree is drawn to scale; the scale bar indicates nucleotide substitutions (%) per site. The isolate of *R. vini* n. sp. was obtained from the tick *Ixodes arboricola* (Czech Republic).

Legend: GenBank accession number, rickettsial species, source of the isolate and country.


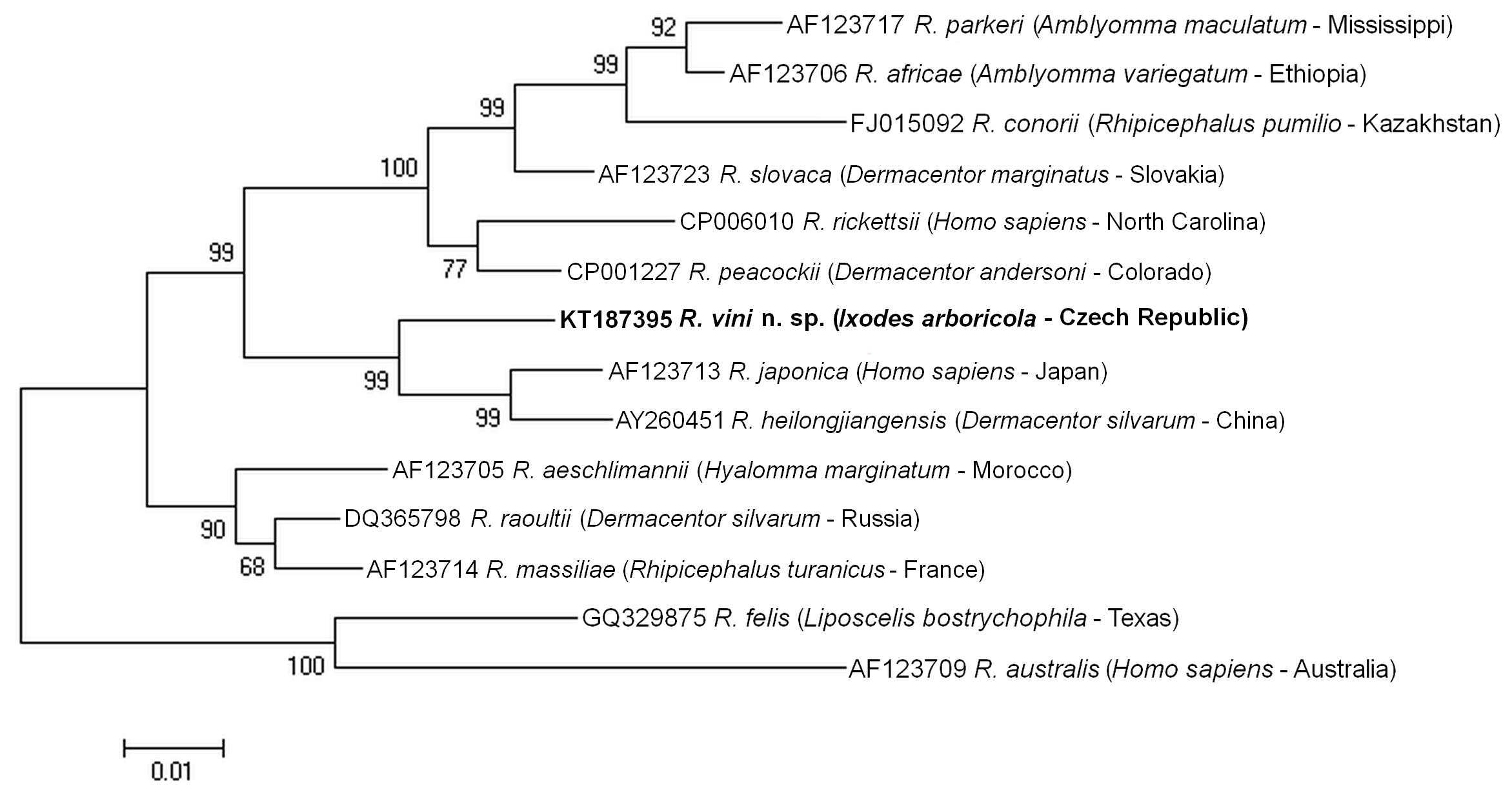

Supplement: Additional file 3: — Maximum Likelihood phylogenetic tree based on the partial ompB gene including a sequence for Rickettsia vini n. sp. (DOCX 639 kb) [file 13071_2016_1742_MOESM3_ESM.docx]
